# Cork-in-Bottle Occlusion of Fluoride Ion Channels by Crystallization Chaperones

**DOI:** 10.1016/j.str.2018.02.004

**Published:** 2018-04-03

**Authors:** Benjamin C. McIlwain, Simon Newstead, Randy B. Stockbridge

**Affiliations:** 1 Program in Biophysics, University of Michigan, Ann Arbor, MI 48109, USA; 2 Department of Biochemistry, University of Oxford, Oxford OX1 3QU, UK; 3 Department of Molecular, Cellular, and Developmental Biology, University of Michigan, Ann Arbor, MI 48109, USA

**Keywords:** crystallization chaperone, monobody, ion channel

## Abstract

Crystallization of dual-topology fluoride (Fluc) channels requires small, soluble crystallization chaperones known as monobodies, which act as primary crystal lattice contacts. Previous structures of Flucs have been solved in the presence of monobodies that inhibit fluoride currents in single-channel electrophysiological recordings. These structures have revealed two-fold symmetric, doubly bound arrangements, with one monobody on each side of the membrane. The combined electrophysiological and structural observations raise the possibility that chaperone binding allosterically closes the channel, altering the structure from its conducting form. To address this, we identify and solve the structure with a different monobody that only partially blocks fluoride currents. The structure of the channel-monobody complex is asymmetric, with monobody bound to one side of the channel only. The channel conformation is nearly identical on the bound and uncomplexed sides, and to all previously solved structures, providing direct structural evidence that monobody binding does not induce local structural changes.

## Highlights

• Inhibitory crystallization chaperones bind on each side of the membrane
• Crystal structure of channel with one side free of chaperone binding
• S8 chaperone-bound channel is competent for fluoride transport
• Chaperones inhibit channels via a “cork-in-bottle” mechanism of physical occlusion

## Graphical Abstract

**Figure:**
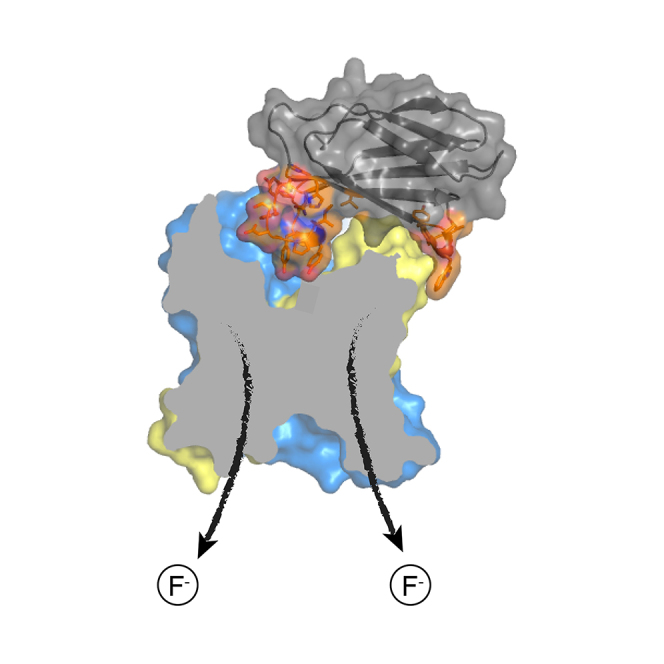

## Teaser

McIlwain et al. present the structure of a fluoride channel bound to an inhibitory chaperone (monobody) on one side. Comparing to previous structures with chaperones on both sides, the authors show that monobody binding does not affect the structure of the channel, likely using physical occlusion to block the channel.

## Introduction

Crystallization of membrane proteins can in many cases be facilitated and diffraction quality improved by complexation with "crystallization chaperone" proteins such as antibody fragments or engineered protein domains (Bukowska and Grutter, 2013). By binding specifically to detergent-solubilized membrane proteins, chaperones increase the protein's aqueous-exposed area and thereby enhance the chances for productive crystal contacts. However, this maneuver, if successful, comes with a price: the possibility that the structure of the protein target differs with and without the chaperone being bound. If achieving a crystal structure requires using a chaperone, how can we know that the structure represents its uncomplexed, biologically relevant conformation? Settling this question requires additional evidence, such as functional experiments (Uysal et al., 2009) or crystallization without chaperones (Jiang et al., 2003, Lee et al., 2005).

**Figure 1:**
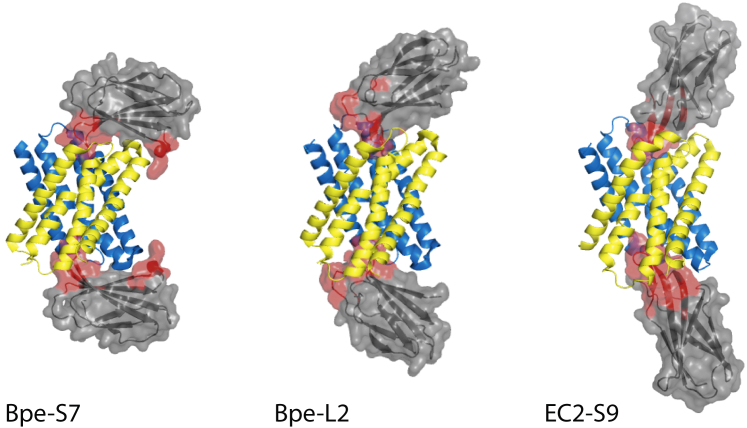
Monobody-Channel Complexes Cartoon representation of Bpe-S7, Bpe-L2, and Ec2-S9 complexes. Channel subunits shown in blue and maize, monobodies in gray with surface rendering. The diversified region of monobody is colored red.

This is particularly problematic in recent crystal structures of the "Fluc" family of fluoride-specific ion channels (Last et al., 2016, Last et al., 2017, Stockbridge et al., 2015). These channels are unusual in that they assemble as symmetrical homodimers with the two subunits arranged in antiparallel transmembrane topology, such that the channels' intracellular and extracellular ion entryways are structurally identical (Stockbridge et al., 2013, Stockbridge et al., 2014, Stockbridge et al., 2015). Moreover, the channel dimer contains two F^−^ permeation pathways, with each subunit contributing residues to both pathways (Last et al., 2016, Last et al., 2017, Stockbridge et al., 2015). The chaperones required to obtain Fluc crystals (engineered "monobodies" derived from a human fibronectin domain and selected from diversified phage-display libraries; Koide et al., 2009, Koide et al., 2012) bind to both ends of the channel protein as in the crystal structures of Figure 1. All previously published structures of Fluc channels, representing four different channel/monobody complexes, show two monobodies bound per channel, one on each end, apparently occluding the wide aqueous vestibules through which ions enter and leave the F^–^-selective transport pathways (Last et al., 2016, Last et al., 2017, Stockbridge et al., 2015, Turman et al., 2018). This doubly bound state is consistent with electrophysiological recordings, which show that, upon binding from either side, these monobodies render the channels nonconductive to F^–^ ions, an inhibitory process commonly called "block" (Stockbridge et al., 2014, Turman et al., 2015, Turman and Stockbridge, 2017).

Our purpose in solving these structures is to gain insight into the hyperselectivity of Fluc channels, which pass F^–^ ions >10^4^-fold more rapidly than the close analog Cl^–^ (Stockbridge et al., 2013). However, in using the structures in this way, we make an implicit assumption: that the monobody-complexed channel is structurally identical to its ion-conducting conformation without chaperones bound. This is a soft assumption; it is possible that monobody binds to an alternate conformation of the channel with closed ion-conduction pathways. If such an allosteric mechanism underlies monobody block, rather than simple cork-in-bottle pore-occlusion, our observed crystal structures would not provide a valid template for examining the F^–^-conducting channel mechanistically. Ideally, this problem would be resolved by a Fluc crystal structure without monobody present, but we have not been able even to crystallize this protein without chaperone complexation. In this report, we describe an *in meso* crystal structure of a Fluc channel with monobody bound to only one end of the conduction pathway. Although this breaks the two-fold transmembrane symmetry of the entire complex, the channel itself remains symmetrical and identical in structure to the previous doubly occupied complexes. We couple our structural observations with electrophysiological recordings that support our conclusion that chaperone binding does not alter the structures of the fluoride channels.

## Results

**Figure 2:**
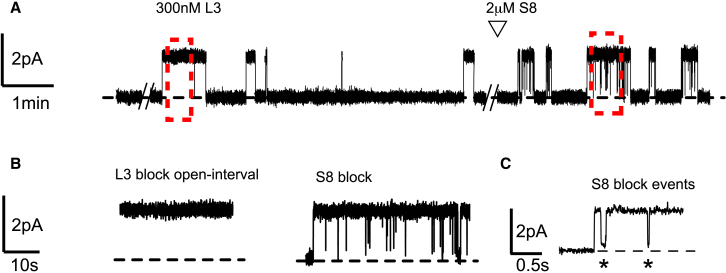
Single-Channel Recording of Bpe with Monobodies L3 and S8 (A and B) (A) Single channels were initially recorded in the presence of 300 nM L3, with S8 added at the indicated time. The zero-current level is indicated by the dashed line. Dashed boxes indicate zoomed-in views shown in (B). (C) Enlarged trace of S8 block events, indicated by asterisks. The fully blocked level (dashed line) is set by L3 block event, left side of trace. See also Table S1.

Of the eight monobodies previously selected as binders against two bacterial Fluc homologs (Stockbridge et al., 2014), S8, directed against the Bpe homolog, is a particularly weak blocker, with affinity in the ∼200 μM range, rather than the sub-micromolar affinity of previously described monobodies (Stockbridge et al., 2014, Stockbridge et al., 2015). This property is illustrated in Figure 2, which shows single-channel recordings of S8 block. The blocker at 2 μM induces very short-lived (20-ms timescale) interruptions of the otherwise almost always-open channel. These short blocks dramatically contrast with block by monobody L3 (included in these same recordings as an internal standard for the 100% block level), which typically last tens of seconds (Table S1) (Stockbridge et al., 2014, Turman et al., 2015, Turman and Stockbridge, 2017). The electrophysiological behavior of S8 is indistinguishable in the presence and absence of L3. Unlike L3, which completely blocks fluoride current upon complexation (Stockbridge et al., 2014), S8 binding events only partially block ion throughput, with residual currents ∼10% of the open-channel conductance (Figure 2C).

**Figure 3:**
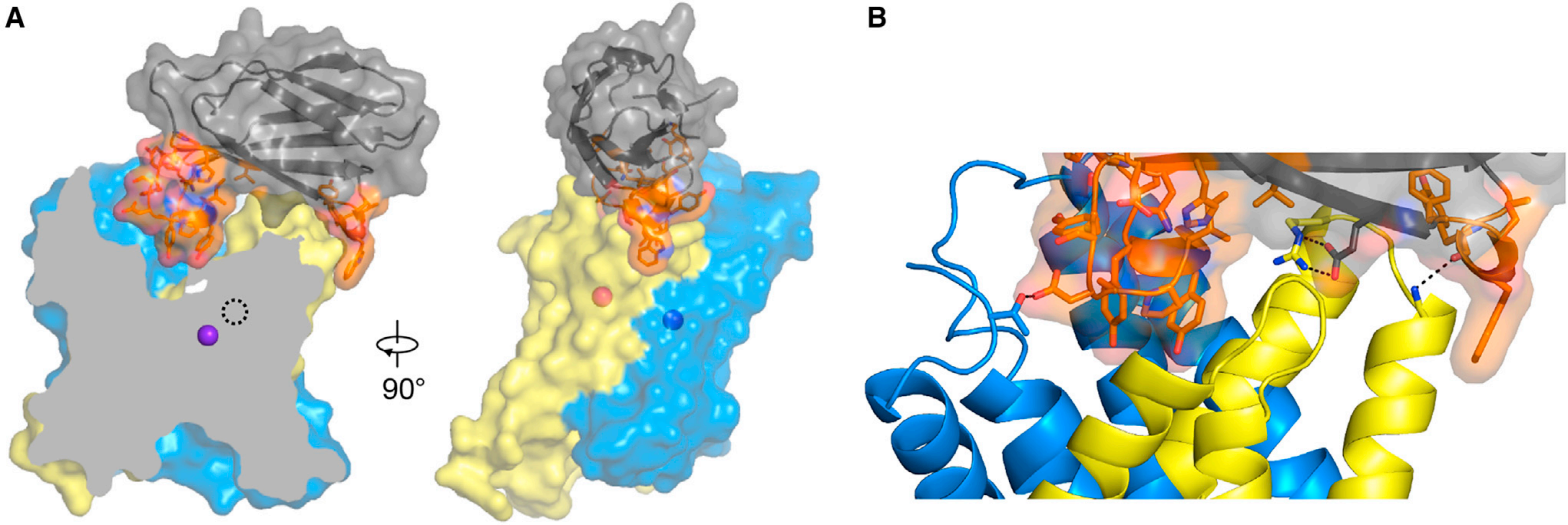
Bpe-S8 Structure Coloring as in Figure 1. (A) Surface and cartoon representation of Bpe-S8 complex. In the leftmost view, the channel is clipped along a plane perpendicular to the membrane in order to show the details of monobody binding in the vestibule. Fluoride ions are shown as spheres, and clipped ion is shown as dashed circle. The monobody's diversified loops are shown in orange. (B) Close-up view of Bpe-S8 interface. Sidechains of diversified loop, and sidechains involved in S8/Bpe H-bond interactions, shown as sticks. Putative H-bonds indicated with dashed lines. From left to right: T3 (Bpe)-D80 (S8); R95 (Bpe)-E48 (S8); Y98 (Bpe backbone); and Y43 (S8 backbone).

*In meso* crystals of the Fluc homolog Bpe in complex with monobody S8 diffracted to 2.8 Å Bragg spacing, and the structure was solved using molecular replacement with the Bpe channel and L2 monobody (Stockbridge et al., 2015) as search models (Table 1). To our surprise, the asymmetric unit contains the dimeric channel with only a single monobody bound, as shown in Figure 3A. One end of the channel has a monobody bound in a familiar way, with its diversified loop occluding the aqueous vestibule, while the other end is free of monobody. A close-up view of the Bpe-S8 monobody interface is illustrated in Figure 3B. The ∼950 Å^2^ interface is mainly hydrophobic. Hydrogen bonds and salt bridges between S8 and the channel are rare and peripheral. The only polar monobody/channel interactions within H-bonding distance are between D80 (S8) and T3 (channel) at the periphery, the carbonyl oxygen of Y43 (S8) and the backbone amide of Y98 (channel) at the opposite periphery, and a salt bridge between E48 (S8) and R95 (channel). The variable loop's polar residues appear to be solvated by bulk water in the vestibule. This dearth of putative H-bonding interactions between S8 and Bpe is a marked departure from previously observed Bpe/monobody interfaces, which typically involve six or seven H-bonded or salt bridge pairs, many within the vestibule, that contribute substantially to the binding affinity (Turman and Stockbridge, 2017, Turman et al., 2018).

**Table 1:** Data Collection, Phasing, and Refinement Statistics

	Bpe-S8

**Data Collection**	

Space group	C 1 2 1
Cell dimensions	
*a*, *b*, *c* (Å)	114.92, 39.85, 109.87
α, β, γ (°)	90, 107.39, 90
Resolution (Å)	27.6–2.8 (2.9–2.8)
*R*_merge_	0.1626 (1.527)
R-pim	0.0605 (0.5842)
Mn *I*/σ*I*	11.68 (1.23)
CC(1/2)	0.997 (0.531)
Wilson B-factor	65.81

**Refinement**	

Resolution (Å)	27.6–2.8 (2.9–2.8)
No. of reflections	11,985 (1,170)
*R*_work_	0.2201 (0.3420)
*R*_free_	0.2752 (0.4089)
Ramachandran favored (%)	94.75
Ramachandran allowed (%)	4.66
Ramachandran outliers (%)	0.58
Clashscore	14.82
RMS (bonds)	0.002
RMS (angles)	0.42

Statistics for the highest-resolution shell are shown in parentheses. RMS, root-mean-square.

**Figure 4:**
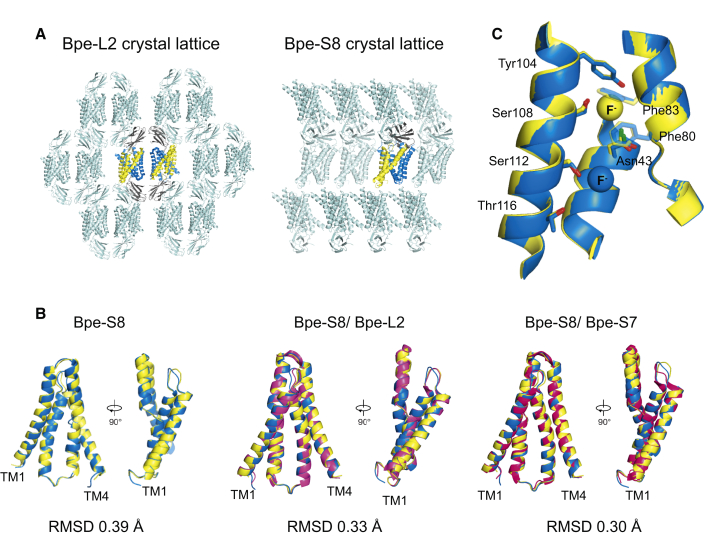
Crystal Lattices and Structural Symmetry of Singly and Doubly Bound Bpe-Monobody Complexes (A) Bpe-L2 lattice (left) and Bpe-S8 crystal lattice (right). The asymmetric unit is shown in maize and blue (channel), and gray (monobody). Symmetry mates shown in cyan. (B) Alignment of Bpe-S8 subunit A (maize) and subunit B (blue). Bpe-S8 A/B overlay aligned with Bpe-L2 subunit A (hot pink) and Bpe-S8 A/B overlay aligned with Bpe-S7 subunit A (magenta). (C) Alignment of Bpe-S8 subunits with polar track residues shown as sticks. F^−^ ions are represented as spheres. Colored as in (B).

The crystal lattice also deviates notably from previously solved Fluc complexes. Whereas normally crystal contacts are mediated almost exclusively by monobodies, in the Bpe-S8 structure the other end of the channel contacts a symmetry-related channel in a back-to-back arrangement (Figure 4A). Despite the asymmetry of the complex, the channel itself retains the same structural symmetry as in the doubly bound channel structures, as shown by aligning one channel subunit (subunit A) of the Bpe-S8 complex with its partner (subunit B) within the same complex (Figure 4B, left). Only a minor departure from strict symmetry of the two channel subunits is seen where the crystal contacts differ, in the short sequence connecting transmembrane helices 1 and 2; the largest backbone deviation in this loop is only 4 Å. Elsewhere, the backbones of the two subunits, and even the side chains, align precisely. Moreover, Bpe in the single-monobody S8 complex aligns well with the same channel in a doubly blocked L2 complex (Figure 4B, middle) and with a different doubly blocked S7 complex (Figure 4B, right). We do not observe any major changes in sidechain rotamers that can be distinguished at 2.8 Å resolution between the two Fluc subunits in the Bpe-S8 complex. An alignment of residues so far implicated in fluoride permeation is shown in Figure 4C; we also do not observe notable differences in the rotamers of any conserved sidechains or sidechains lining the protein's aqueous vestibules. In other words, the conformation of the singly complexed channel is essentially identical to previously described crystal structures in various doubly blocked complexes.

## Discussion

The question driving this work is: do our previous crystal structures of Fluc channels, which have all been of complexes with two monobodies binding per channel, represent the F^–^-conducting conformation? Or does monobody binding, which blocks F^–^ permeation, somehow alter the channel structure to close the pore? A comparison of crystal structures of channels with and without crystallization chaperones to answer this question directly is currently out of reach. We can distinguish two distinct classes of monobody-driven allosteric changes that might occur: a local alteration that closes the conduction pathway near each occupied monobody site and a global change that affects the entire channel symmetrically upon binding of the first monobody. We argue below that both possibilities are ruled out.

First, recent work compared block by monobodies from one side of the channel or simultaneously from both sides (Turman et al., 2015, Turman and Stockbridge, 2017). One such monobody analyzed in detail showed independent binding to the two sites, with quantitatively identical association and dissociation rate constants of the first and second binding event; a compelling refutation of a global allosteric change whereby binding the first monobody alters the channel structure symmetrically on both sides. Such a picture predicts positive cooperativity for the two binding events. However, independent binding of the two blockers would still be consistent with a local allosteric mechanism, where the channel's pore structure is altered only near the site of each monobody binding, while remaining unchanged at the other end of the pore. However, the Bpe-S8 crystal structure directly rules out this idea. Although only one end of the channel binds a monobody, both ends are structurally identical, as well as identical to all published doubly complexed structures. In addition, strict geometric constraints imposed by H-bonding or salt bridge interactions are notably absent in the vestibule, further undermining the notion of a local allosteric effect near the polar fluoride conduction pathways.

Electrophysiological recording of monobody block further supports this picture. While all eight monobodies selected to bind to our Fluc homologs are channel blockers (Stockbridge et al., 2014), neither S8 nor S7 (Stockbridge et al., 2015) block fully. In both cases, the partial block events reduce F^–^ throughput to ∼10%–15% of the open rate without monobody. This residual conductance represents a robust throughput of ∼900,000 F^–^ ions per second, well within the range expected for conventional channel-mediated electrodiffusion. And yet, as we have seen above, the channel in the Bpe-S7 and Bpe-S8 crystal structure aligns precisely with the channel in complex with monobody L2, which blocks F^−^ current completely. For these reasons, the Bpe-S8 structure, taken together with the electrophysiological results, provides strong support for a cork-in-bottle mechanism of monobody block and its logical accompaniment: that the crystal structures of Fluc channels represent the functional, F^–^-conducting conformation of the protein.

## STAR★Methods

### Key Resources Table

**Table:** 

REAGENT or RESOURCE	SOURCE	IDENTIFIER
**Chemicals, Peptides, and Recombinant Proteins**		

S8 monobody recombinant protein	Stockbridge et al., 2014	N/A
Fluoride channel from B. pertussis recombinant protein	Stockbridge et al., 2013	N/A

**Deposited Data**		

Structure of a dual topology fluoride channel with monobody S8	This paper	PDB: 6BQO

**Recombinant DNA**		

pET21c with Bpe gene, C-terminal hexahistidine tag	Stockbridge et al., 2013	N/A
pHFT2 with S8 gene, N-terminal histidine tag	Stockbridge et al., 2014	N/A

**Software and Algorithms**		

PHASER	McCoy et al., 2007	http://www.ccp4.ac.uk/html/phaser.html
BUSTER	Blanc et al., 2004	
Coot	Emsley et al., 2010	https://www2.mrc-lmb.cam.ac.uk/personal/pemsley/coot/

### Contact for Reagent and Resource Sharing

Further information and requests for resources and reagents should be directed to and will be fulfilled by the Lead Contact, Randy Stockbridge (stockbr@umich.edu)

### Method Details

Expression in *E*. *coli* and purification of the Fluc homologue "Bpe" from *B. pertussis* and monobody "S8" were as described in detail (Stockbridge et al., 2013, Stockbridge et al., 2014). *In meso* crystallization of the Bpe-S8 complex was carried out as with the Bpe-L2 complex (Stockbridge et al., 2015), with crystals appearing in 2-3 days in several low molecular weight PEGs, including 30% PEG 400, 550MME and 600. Final, optimized crystals were grown in 26% PEG 550MME, 0.1M sodium citrate, pH 5.0 and diffraction data collected at beamline I04 Diamond Light Source, UK. Phases were calculated following molecular replacement with PHASER (McCoy et al., 2007), employing the previous Fluc channel structure (PDB: 5NKQ) and a trimmed structure of the S7 monobody (PDB: 5A40) with the loop regions removed. The Bpe-S8 model was built into the electron density maps calculated from BUSTER (Blanc et al., 2004), following iterative rounds of structure refinement. The structural model was revised and built in real space with Coot (Emsley et al., 2010).

For single-channel recording in planar phospholipid bilayers, purified Fluc channel protein was reconstituted into liposomes (*E*. *coli* polar lipids) at 0.05 μg/mg lipid, as described (Stockbridge et al., 2013, Turman and Stockbridge, 2017). Cis- and trans- chambers contained 15 mM MOPS, pH 7, 300 mM NaF, and 50 μg/mL bovine serum albumin, and temperature was maintained at 23-24°C. Recordings were acquired at -200 mV holding voltage, electronically filtered at 1 kHz during acquisition, and digitally filtered to 100-500 Hz for analysis. Dwell time kinetics were determined from single-exponential fits to cumulative distribution histograms of open and blocked intervals, consisting of ∼20-200 events. Kinetic parameters were estimated according to a bimolecular block scheme as described (Stockbridge et al., 2014):

(Equation 1) 1/τ_O_ = k_on_ [M]

(Equation 2) 1/τ_B_ = k_off_

### Quantification and Statistical Analysis

Reported values represent the mean and SEM of 3 independent runs in separate bilayers.

### Data and Software Availability

The coordinates for the X-ray crystal structure of Bpe-S8 have been deposited in the PDB under ID code 6BQO.

## Supplemental Information

Table S1. Time and Rate Constants for Monobody Block for Data Presented in Figure 2

Document S2. Article plus Supplemental Information
