# Supplementary material for: Cork-in-Bottle Occlusion of Fluoride Ion Channels by Crystallization Chaperones
Source: Structure. 2018 Apr 3;26(4):635–639.e1. doi: 10.1016/j.str.2018.02.004 (PMC5884710; doi:10.1016/j.str.2018.02.004)
Supplement: Table S1. Time and Rate Constants for Monobody Block for Data Presented in Figure 2 [file mmc1.pdf]

Structure, Volume *26*

Supplemental Information

Cork-in-Bottle Occlusion of Fluoride Ion Channels by Crystallization Chaperones

Benjamin C. McIlwain, Simon Newstead, and Randy B. Stockbridge

Supplemental Kinetic Data for Figure 2

Table S1. Time and rate constants for monobody block

	τ_B (sec)	k_{off} (s^{-1})	τ_O (sec)	k_{on} (s^{-1} M^{-1})	K_d
L3	19.6±0.10	0.05	9.4±0.022 (with 300 nM Mb)	3.5 x 10^5	135 nM
S8	0.021 ±0.003	47.6	2.0±0.6 (with 2 μM Mb)	2.5 x10^5	190 μM
